# PTEN-mediated resistance in cancer: From foundation to future therapies

**DOI:** 10.1016/j.toxrep.2025.101987

**Published:** 2025-03-04

**Authors:** Muhammad Tufail

**Affiliations:** Department of Oral and Maxillofacial Surgery, Center of Stomatology, Xiangya Hospital, Central South University, Changsha, China

**Keywords:** PTEN, Cancer, Drug resistance, Signaling pathways, Therapeutic approaches

## Abstract

In cancer resistance, *phosphatase and tensin homolog deleted* (PTEN) has emerged as a prominent protagonist. *PTEN* exerts its influence by regulating crucial signaling pathways that govern cell proliferation, survival, and differentiation. This comprehensive review article investigates deeply into the complex realm of *PTEN*-mediated drug resistance mechanisms in cancers. Our journey begins by exploring *PTEN's* foundational role of PTEN, unveiling its significance as a molecular conductor that intricately coordinates vital cellular pathways. We thoroughly dissected the intricate milieu of *PTEN* alterations, including mutations, deletions, and epigenetic silencing, and elucidated their profound implications for fueling cancer growth and evading treatment. As we navigate the complex network of *PTEN*, we unravel the intricate interplay between *PTEN* and pivotal signaling pathways, such as PI3K/AKT, MAPK/ERK, and Wnt/β-catenin, further complicating the resistance landscape. This expedition, through these intricately intertwined signaling cascades, provides insight into the multifaceted mechanisms driving resistance, thereby revealing potential exploitable weaknesses. In our quest for therapeutic strategies, we need to explore innovative approaches to restore *PTEN* function, encompassing genetic therapies, pharmacological agents, and precision medicines tailored to *PTEN* status. The concept of combination therapy has emerged as a potent tool to overcome *PTEN*-associated resistance, offering promising synergistic interactions with standard treatments, targeted therapies, or immunotherapy. This review offers a comprehensive overview of *PTEN*-mediated drug resistance mechanisms in cancer and elucidates intricate interactions within this complex landscape. This underscores the central role of *PTEN* in drug resistance and provides valuable insights into promising strategies with the potential to reshape the future of cancer treatment.

## Highlights

• PTEN, a central tumor suppressor, plays a pivotal role in cancer drug resistance.
• PTEN is a foundational molecular conductor, intricately coordinating essential cellular pathways.
• Delving into PTEN mutations, deletions, and epigenetic changes, revealing their profound impact on fueling cancer growth and resisting treatment.
• Navigating the complex interplay between PTEN and crucial signaling pathways, complicating the landscape of drug resistance.
• Exploring innovative strategies, from genetic therapies to precision medicines, to restore PTEN function.
• Emphasizing combination therapy as a powerful approach against PTEN-associated resistance, offering synergies with standard and targeted treatments.

## 1 Introduction

**Fig. 1:**
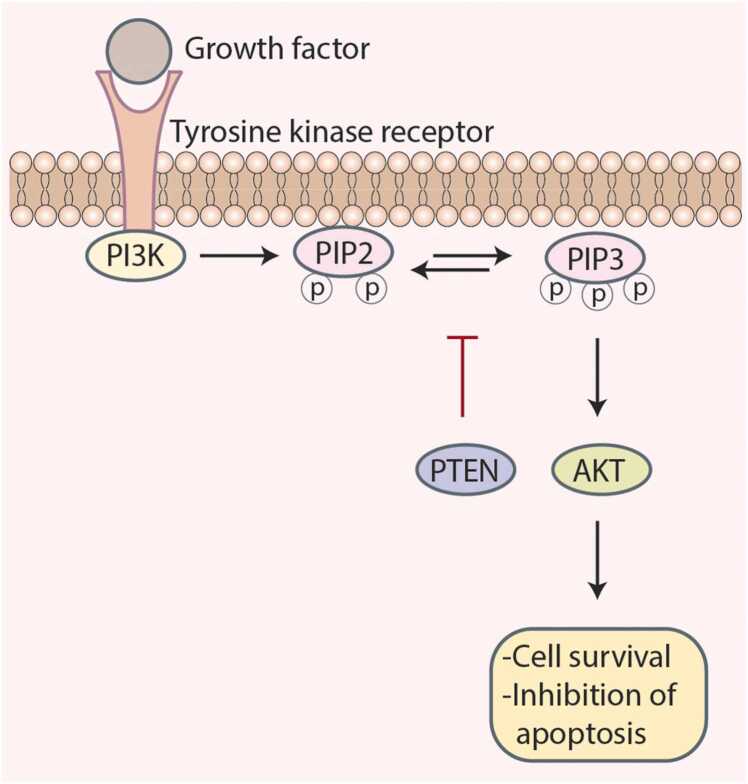
PTEN negatively regulates the PI3K/Akt signaling pathway by dephosphorylating PIP3 back to PIP2, thereby inhibiting Akt activation. This suppression prevents excessive cell survival signaling and promotes apoptosis. Loss or inactivation of PTEN leads to unchecked Akt activation, contributing to cancer progression and resistance to therapy.

Cancer is an intricate and devastating disorder that continues to take a substantial toll on global public health [1], [2]. Its intricacy arises from an intricate web of molecular aberrations that drive unbridled cell proliferation and evade programmed cell death [3], [4]. Amidst this molecular complexity, *PTEN* has emerged as a central, yet enigmatic, protagonist. *PTEN*, a tumor suppressor gene, is pivotal in governing cellular processes including proliferation [5], [6], differentiation [7], [8], and survival (Fig. 1) [9]. Its influence primarily emanates from lipid phosphatase activity, which antagonizes the phosphoinositide 3-kinase (PI3K)/Akt signaling cascade, inhibits cell growth, and facilitates apoptosis.

However, *PTEN's* role in cancer is contradictory and is characterized by a dual nature akin to Janus [10]. PTEN assumes the mantle of a guardian, safeguarding genomic stability, preserving cellular integrity, and persistently opposing oncogenic transformation [11]. In this context, it is unequivocally recognized as a quintessential tumor suppressor. Conversely, PTEN's repertoire transcends its classical role, increasingly emerging as a central player in the intricate phenomenon of resistance to potent cancer therapies [12]. The dual role of PTEN in cancer, as both a tumor suppressor and orchestrator of therapeutic resistance, constitutes the core theme of this comprehensive review.

Understanding PTEN's role of PTEN in cancer requires a clear understanding of its fundamental functions as a tumor suppressor. This includes understanding how PTEN hinders cell growth, prevents blood vessel formation, and supports genetic stability. It is also crucial to explore how PTEN interacts with other cancer-related genes that influence cell behavior. This foundational knowledge is essential for understanding how PTEN contributes to resistance to cancer treatment.

Subsequently, we embarked on an expedition through the burgeoning landscape of PTEN as a mediator of cancer resistance. Herein, we traverse diverse therapeutic domains encompassing targeted therapies, immunotherapies, and conventional cytotoxic agents. We examined how PTEN status modulates responses to treatment, elucidated the mechanisms by which PTEN-deficient cells elude therapeutic assaults, and investigated the clinical development of PTEN-mediated resistance. Furthermore, we explored potential therapeutic strategies to circumvent or control PTEN-related resistance mechanisms, providing a glimpse into the future of precision cancer therapies.

## 2 Molecular mechanisms of *PTEN* loss in cancer

### 2.1 PTEN mutations and deletions

*PTEN* acts as a guardian in the complex molecular environment of cancer, and its loss or inactivation marks a pivotal occurrence in the development of tumors [13], [14]. *PTEN*, a pivotal negative regulator of the PI3K/Akt signaling pathway, modulates cellular growth and survival by counteracting PI3K-dependent phosphorylation of phosphatidylinositol 4,5-bisphosphate (PIP2) to phosphatidylinositol 3,4,5-trisphosphate (PIP3) [15], [16]. This dephosphorylation event is a critical checkpoint for Akt activation during downstream signaling. *PTEN's* tumor-suppressive function of PTEN relies on its capacity to maintain a finely tuned balance of PIP3 levels, thereby restraining aberrant cell growth and proliferation.

However, *PTEN* mutations, frequently encountered in cancer, disrupt this balance [17], [18]. These mutations, including missense, nonsense, frameshift, or splice site mutations, engender loss of *PTEN* function or compromise its enzymatic activity. Consequently, PIP3 levels surge, perpetuating Akt activation and the ensuing downstream signaling cascade [19]. This unrestrained PI3K/Akt signaling promotes cell survival, growth, angiogenesis, and oncogenesis, and renders cancer cells impervious to therapies that induce apoptosis.

Homozygous deletion of *PTEN* is another pivotal mechanism for *PTEN* loss in cancer. These deletions typically encompass both *PTEN* alleles, resulting in the complete *PTEN* inactivation of PTEN [20]. Such occurrences are prevalent in various cancer types, including TNBC [21], bladder cancer [22], and prostate cancer [23]. The absence of functional *PTEN* in cells undergoing homozygous deletion renders them entirely reliant on the activated PI3K/Akt pathway for survival and growth [24]. This dependence on PI3K/Akt signaling fuels tumorigenesis and poses a formidable obstacle for therapeutic interventions. The absence of *PTEN* relies on this pathway, thereby making cancer cells less amenable to therapies targeting alternative signaling routes and culminating in drug resistance.

Heterozygous deletions of *PTEN*, in which only one allele is lost, give rise to *PTEN* haploinsufficiency [20]. In such instances, *PTEN* levels diminish but do not reach complete extinction. This partial loss of *PTEN* function may instigate the activation of the PI3K/Akt pathway, albeit to a lesser extent than full *PTEN* loss [19].

In addition to mutations and deletions that directly affect *PTEN's* coding sequences, genomic alterations in regulatory elements, such as promoter methylation [25], [26] and miRNA dysregulation, may impede *PTEN* expression [27], [28]. These epigenetic modifications can lead to diminished *PTEN* expression at the transcriptional or post-transcriptional level, further contributing to the resistance mechanisms inherent to *PTEN* loss.

Understanding the molecular mechanisms underlying PTEN loss is paramount for predicting treatment responses and designing precise therapies. PTEN-deficient cancers frequently exhibit resistance to therapies that target the PI3K/Akt pathway. Therefore, identifying *PTEN* status in patients can guide treatment decisions, inform therapeutic choices, and predict the likelihood of drug resistance.

### 2.2 Epigenetic regulation of PTEN expression

Epigenetic changes play a central role in governing PTEN levels and significantly contribute to the development of resistance to various cancer treatments [29]. DNA methylation is a fundamental epigenetic mechanism regulating gene expression. In cancer cells, the promoter region of PTEN can experience hypermethylation, resulting in the suppression of PTEN expression [30]. Hypermethylation of CpG islands within the PTEN promoter region impedes the binding of transcription factors and RNA polymerase to the promoter region, thereby effectively obstructing PTEN transcription [31]. This epigenetic alteration represents a potent mechanism through which PTEN expression is negatively regulated in cancer cells. The resultant decrease in PTEN levels triggers constitutive activation of the PI3K/Akt signaling pathway, facilitating cell survival and conferring resistance to apoptosis-inducing therapies.

MicroRNAs (miRNAs) are small non-coding RNA molecules that regulate gene expression post-transcriptionally by binding to the 3′ untranslated region (UTR) of the target mRNA [32]. Several miRNAs have been identified that directly target *PTEN* mRNA for degradation or translational inhibition [33]. In cancer, dysregulated miRNA expression can lead to increased levels of miRNAs that target *PTEN*. This results in decreased *PTEN* protein levels, even in the presence of *PTEN* mRNA, thereby promoting oncogenic signaling through the PI3K/Akt pathway. The downregulation of specific miRNAs, such as miR-21 and miR-22, has been implicated in *PTEN* downregulation and drug resistance in various cancer types [34], [35].

Several miRNAs have been identified that either upregulate or downregulate PTEN expression through distinct mechanisms, thereby impacting disease progression. For instance, miR-21 has been reported to downregulate PTEN expression by directly targeting its mRNA in gastric cancer, head and neck squamous cell carcinoma (HNSCC), and clear cell renal cell carcinoma (CCRCC) [36], [37]. Similarly, miR-130 exhibits both upregulation and downregulation of PTEN expression in various diseases. In bladder cancer, breast invasive carcinoma, human coronary artery endothelial cell (HCAECs) injury, inflammatory responses, Parkinson's disease (PD), lung adenocarcinoma, and colon adenocarcinoma, miR-130 downregulates PTEN by directly targeting its mRNA [38], [39]. Conversely, in non-small cell lung cancer (NSCLC), miR-130 upregulates PTEN expression through direct mRNA targeting [40].

Additionally, miR-451 upregulates PTEN expression by directly targeting its mRNA in lung cancer and ovarian cancer [41], [42]. Conversely, miR-221/222 downregulates PTEN expression by directly targeting PTEN mRNA in diseases such as NSCLC, hepatocellular carcinoma (HCC), and TNF-related apoptosis-inducing ligand (TRAIL)-induced cell death [43].

Furthermore, miRNAs such as miR-301a [44], [45], miR-214 [46], [47], miR-494 [48], [49], and a cluster including miR-155–5p, miR-130b, miR-616, miR-19, miR-92a, miR-10a, miR-106a, miR-429, miR-26a, and miR-486–5p [50], [51] downregulate PTEN expression by directly targeting its mRNA in various cancers and other diseases.

Moreover, certain miRNAs, such as miR-29 [52], [53], miR-101[54], [55], and miR-185 [56], upregulate PTEN expression by inducing hypomethylation of the PTEN promoter through inhibition of DNA methyltransferase (DNMTs) expression. This regulation occurs in diseases, such as liver fibrosis, lung cancer, and HCC. The association between miRNAs, lncRNAs, and PTEN expression is shown in Table 1 [57].

**Table 1:** Regulation of PTEN expression by miRNAs and lncRNAs.

**MiRNA**		**PTEN expression**		**Mechanism**		**Disease**			**Reference**
miR−21		Down		directly targeting PTEN mRNA		Gastric cancer, HNSCC, CCRCC			[36], [37]
miR−130		Down		directly targeting PTEN mRNA		Bladder cancer, Breast invasive carcinoma, HCAECs injury, Inflammatory responses, PD, Lung adenocarcinoma, Colon adenocarcinoma			[38], [39]
miR−130		Up		directly targeting PTEN mRNA		NSCLC			[40]
miR−451		Up		directly targeting PTEN mRNA		Lung cancer, Ovarian cancer			[41], [42]
miR−221 /222		Down		directly targeting PTEN mRNA		NSCLC,HCC, TRAIL-induced cell death			[43]
miR−301a		Down		directly targeting PTEN mRNA		Breast cancer, Neuronal death, Ewing’s carcoma, Melanoma, Insulin resistance			[44], [45]
miR−214		Down		directly targeting PTEN mRNA		Tumorigenesis, Immunology, Cardiac injury			[46], [47]
miR−494		Down		directly targeting PTEN mRNA		Ischemia/Reperfusion -induced myocardial injury			[48], [49]
miR−155–5p/130b/ 616/19/92a/10a/ 106a/429/26a /486–5p		Down		directly targeting PTEN mRNA		HCC, NSCLC, Breast cancer, Lung cancer, Colorectal Cancer, Chronic myeloid leukemia, Intestinal cancer, Acute T-cell lymphoblastic leukemia			[50], [51]
miR−29		Up		inducing the hypomethylation of PTEN promoter by inhibiting DNMT1, DNMT3b and SET1A expression		Liver fibrosis			[52], [53]
miR−101		Up		inducing the hypomethylation of PTEN promoter by inhibiting DNMT3A expression		Lung cancer			[54], [55]
miR−185		Up		inducing the hypomethylation of PTEN promoter by inhibiting DNMT1 expression		HCC			[56]
**LncRNA**	**PTEN expression**		**Mechanism**		**MiRNA**		**Disease**	**Reference**	
PTENP1	Up		acting as ceRNAs		miR−21, miR−17, miR−214, miR−19, miR−20, miR−93, miR−106b, miR−26		CCRCC, OSCC, HCC, Gastric cancer	[37], [58], [59]	
HOTAIR	Up		acting as ceRNAs		miR−19		Cardiac hypertrophy, CRC	[60], [61]	
Linc-USP16	Up		acting as ceRNAs		miR−21,miR−590–5p		HCC	[62]	
LncRNA-BGL3	Up		acting as ceRNAs		miR−17, miR−20a, miR−20b, miR−93, miR−106a		Chronic myeloid leukemia	[63]	
CASC2	Up		acting as both ceRNAs and downregulators of miRNAs		miR−21, miR−181a		Osteosarcoma, Glioma, Cervical cancer	[64], [65]	
MEG3	Up		acting as both ceRNAs and downregulators of miRNAs		miR−1297, miR−19a, miR−21		Breast cancer, Glioma, CAD	[66], [67]	
lncRNA GAS5	Up		acting as both ceRNAs and downregulators of miRNAs		miR−21, miR−103, miR−196a, miR−205, miR−32–5p		HER2-positive breast cancer, HCC, NSCLC, Cardiac fibrosis, Endometrial cancer, Cervical cancer, Pancreatic cancer	[68], [69]	
XIST	Up		acting as both ceRNAs and downregulators of miRNAs		miR−181a, MiR−494		HCC, Spinal Cord Injury	[70], [71]	
NBAT1	Up		acting as both ceRNAs and downregulators of miRNAs		miR−21		Osteosarcoma	[72]	
lnc−2 /lnc−6	Up		acting as both ceRNAs and downregulators of miRNAs		miR−26a		Prostate cancer, Glioma	[73], [74]	
FER1L4	Up		acting as both ceRNAs and downregulators of miRNAs		miR−106a−5p		Colon cancer, Gastric cancer	[75], [76]	
lincRNA-p21	Up		acting as both ceRNAs and downregulators of miRNAs		miR−181b		Liver fibrosis	[77]	
PTENP1 asRNA β	Up		increasing the stability and miRNA sponge activity of PTENP1		–		–	[78]	
HOTAIR	Down		enhancing PTEN methylation via miRNA sponging		miR−29b		Liver Fibrosis, LSCC	[79], [80]	
PTENP1 asRNA α	Down		enhancing PTEN methylation via the recruitment of DNMT3a and EZH2		–		–	[78]	

Furthermore, several long non-coding RNAs (lncRNAs) have been implicated in the regulation of PTEN expression through various mechanisms involving microRNAs (miRNAs), which contribute to the pathogenesis of different diseases. PTENP1, for instance, acts as a competing endogenous RNA (ceRNA) to upregulate PTEN expression by sequestering miRNAs such as miR-21, miR-17, miR-214, miR-19, miR-20, miR-93, miR-106b, and miR-26 in clear cell renal cell carcinoma (CCRCC), oral squamous cell carcinoma (OSCC), hepatocellular carcinoma (HCC), and gastric cancer [37], [58], [59]. Similarly, HOTAIR [60], [61], Linc-USP16 [62], LncRNA-BGL3 [63], CASC2 [64], [65], MEG3 [66], [67], lncRNA GAS5 [68], [69], XIST [70], [71], NBAT1 [72], lnc-2/lnc-6 [73], [74], FER1L4 [75], [76], and lincRNA-p21 [77], exert regulatory effects on PTEN expression through ceRNA mechanisms. For instance, HOTAIR acts as a ceRNA to upregulate PTEN by sponging miR-19 during cardiac hypertrophy. Conversely, HOTAIR downregulates PTEN by enhancing PTEN methylation via miR-29b sponging in liver fibrosis and laryngeal squamous cell carcinoma (LSCC). PTENP1 asRNA β increases PTENP1 stability and miRNA sponge activity without directly interacting with miRNAs, while PTENP1 asRNA α downregulates PTEN by recruiting DNA methyltransferase 3a (DNMT3a) and enhancer of zeste homolog 2 (EZH2). These lncRNAs are critical regulators of PTEN expression in various diseases, highlighting their potential as therapeutic targets for modulating the PTEN signaling pathway.

Histone modifications, including acetylation, methylation, phosphorylation, and ubiquitination, can dynamically modulate chromatin structure and gene expression [81]. Epigenetic alterations in histone marks around the *PTEN* locus can profoundly influence *PTEN* expression. For instance, the loss of the H3K27ac histone mark at the *PTEN* promoter region downregulates *PTEN* expression in glioblastoma cells [82]. Similarly, loss of the H3K4me3 histone mark at the *PTEN* promoter region downregulates *PTEN* expression in prostate cancer cells [83]. Moreover, repressive histone marks such as histone deacetylases (HDACs) and histone methyltransferases (HMTs) can induce chromatin compaction and transcriptional repression of *PTEN*. Conversely, the removal of repressive marks and addition of activating histone marks can facilitate *PTEN* transcription [84].

Understanding the epigenetic regulation of *PTEN* expression is of paramount importance in cancer therapy. Epigenetic alterations leading to PTEN downregulation of *PTEN* can profoundly impact treatment responses and contribute to drug resistance [85], [86]. Therapeutic strategies aimed at reversing the epigenetic silencing of *PTEN* are actively being investigated in clinical research. These approaches include the utilization of DNA demethylating agents, histone deacetylase inhibitors, and miRNA-targeting strategies to restore *PTEN* expression and sensitize cancer cells to treatment.

### 2.3 Post-translational modifications of PTEN

Post-translational modifications (PTMs) are chemical changes that occur in proteins after ribosome synthesis. PTMs can affect the structure, function, localization, and interactions of proteins, and play important roles in various biological processes. Phosphorylation is a pivotal PTM that regulates *PTEN* function of PTEN. In its active state, *PTEN* counters the PI3K/Akt signaling pathway by dephosphorylating PIP3 into PIP2. However, *PTEN* itself can undergo phosphorylation at specific residues such as Ser380, Thr366, and Ser370, potentially modulating its activity.

In cancer, aberrant phosphorylation events can either enhance *PTEN* function, promote tumor suppression, or attenuate its phosphatase activity, thereby reducing its tumor-suppressive potential. Disrupted phosphorylation can lead to *PTEN* inactivation, promoting uncontrolled PI3K/Akt signaling, cell survival, and resistance to therapies targeting this pathway [87].

Ubiquitination is a crucial posttranslational modification that regulates *PTEN* turnover. *PTEN* can undergo ubiquitination primarily at residues Lys13 and Lys289, marking it for proteasomal degradation [88], [89]. E3 ubiquitin ligases such as NEDD4–1 and WWP2 orchestrate this process. Upregulated E3 ligase activity in cancer can expedite *PTEN* degradation, resulting in decreased *PTEN* levels [88]. Ubiquitin-mediated degradation compromises *PTEN's* ability to inhibit PI3K/Akt signaling, thereby promoting carcinogenesis and resistance to therapies targeting the PI3K/Akt pathway.

Acetylation is the addition of an acetyl group to a lysine residue in a protein. *PTEN* acetylation can occur on different lysine residues and has different effects on its activity. For example, acetylation of K125 by p300/CBP enhances *PTEN* activity by increasing its affinity for PIP3 [90], [91]. Acetylation of K402 by Tip60 also inhibits *PTEN* activity by blocking its nuclear localization [92]. Acetylation is emerging as a crucial PTM governing *PTEN's* stability and function. Acetylation of *PTEN* at specific lysine residues such as Lys125 and Lys128 may influence its subcellular localization and activity. In cancer, dysregulated acetylation can affect *PTEN's* subcellular distribution, altering its accessibility to lipid substrates and affecting phosphatase activity. Altered acetylation patterns may contribute to resistance by promoting sustained PI3K/Akt signaling and cell survival.

SUMOylation is a post-translational modification that involves attachment of a small SUMO to another protein. SUMOylation can affect the function, stability, localization, and interactions of target proteins. In cancer, altered SUMOylation patterns can disrupt *PTEN* localization, potentially sequestering it in the cytoplasm and reducing its presence in the plasma membrane [93], [94]. This mislocalization may reduce the effectiveness of *PTEN* in inhibiting PI3K/Akt signaling, thereby contributing to drug resistance [88].

Understanding the impact of PTMs on *PTEN* function and stability is essential for predicting treatment responses and developing precise cancer therapies. Dysregulated PTMs can significantly influence *PTEN*-mediated resistance mechanisms by modulating PI3K/Akt signaling and cell survival.

Ongoing efforts to target the PTMs of *PTEN*, such as modulating phosphorylation, ubiquitination, acetylation, and SUMOylation, have been actively investigated as potential therapeutic strategies. These approaches aim to restore or stabilize *PTEN* function, sensitize cancer cells to treatment, and overcome the resistance mechanisms.

## 3 PTEN and resistance to targeted therapies

### 3.1 PTEN loss and resistance to EGFR inhibitors in lung cancer

**Fig. 2:**
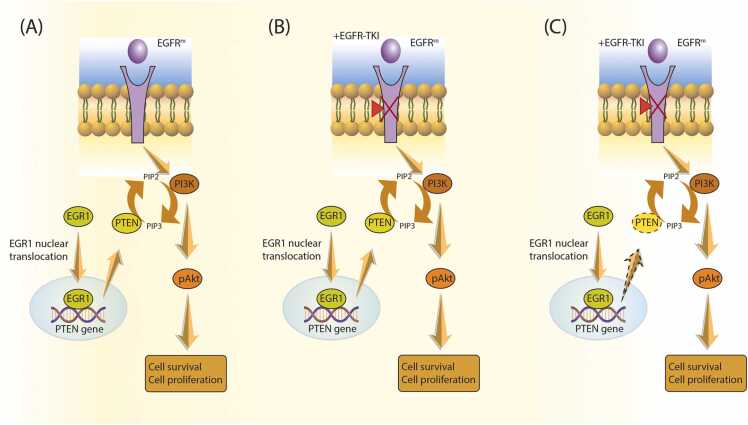
A model illustrates how resistance to EGFR-targeted drugs develops due to the loss of PTEN expression, caused by impaired nuclear translocation of EGR1 in lung cancer cells: **A**. In cells harboring EGFR-activating mutations (EGFRm), the phosphatidylinositol 3-kinase (PI3K)/Akt pathway remains continuously activated, promoting cell survival. PTEN normally acts as a negative regulator of the PI3K/Akt pathway by dephosphorylating PIP3. However, EGFR mutations constitutively activate the PI3K/Akt pathway, leading to sustained cell survival and proliferation. **B**. EGFR-TKI (Epidermal Growth Factor Receptor Tyrosine Kinase Inhibitor) suppresses the activation of EGFR tyrosine kinase, resulting in the inhibition of PI3K/Akt signaling. When EGFR-TKIs are employed to treat lung cancer cells carrying EGFRm mutations, this inhibition of PI3K/Akt signaling contributes to cell death. **C**. However, resistance to EGFR-TKI can develop due to the loss of PTEN expression, leading to further amplification of PI3K/Akt signaling. This acquired resistance allows drug-resistant lung cancer cell lines like PC-9/GEF to persist. Within these resistant cells, PTEN expression is diminished because of impaired translocation of EGR1 to the nucleus.

Although epidermal growth factor receptor (EGFR) inhibitors have revolutionary changes in the treatment landscape of lung cancer, drug resistance remains a formidable clinical challenge, with *PTEN* alterations emerging as pivotal players in this resistance narrative [95], [96]. EGFR inhibitors, epitomized by erlotinib [97], [98], gefitinib [99], [100], and Osimertinib [101], [102], have ushered in a revolutionary transformation in the therapeutic landscape of non-small cell lung cancer (NSCLC). These targeted therapeutics adeptly quell aberrant EGFR signaling, a pervasive aberration caused by EGFR mutations. However, the evolution of EGFR inhibitor resistance remains an enigmatic clinical conundrum. The absence of *PTEN* in lung cancer cells is a pivotal determinant of their resistance to EGFR inhibitors. The intricate interplay between the EGFR and PI3K/Akt pathways is of paramount significance. Without *PTEN*, the regions governing PI3K/Akt signaling are loosened, permitting continuous activation [103], [104]. This protracted activation promotes cell survival, proliferation, and evasion of apoptosis. The sustained activation of PI3K/Akt signaling brought about by *PTEN* loss engenders an environment in which cancer cells become less reliant on EGFR signaling for sustenance and propagation. Consequently, these cells exhibit reduced susceptibility to the growth- and apoptosis-inducing effects of EGFR inhibitors (Fig. 2) [105].

*PTEN*-deficient cells activate alternative signaling cascades, such as the Mitogen-Activated Protein Kinase (MAPK) pathway, to perpetuate their survival and proliferation without EGFR signaling.

In cancer, altered PTEN expression or activity can affect the response to EGFR inhibitors by modulating various signaling pathways that regulate cell survival and apoptosis. For example, *PTEN* loss may render cancer cells less susceptible to the pro-apoptotic effects of EGFR inhibitors by activating the PI3K/AKT and other anti-apoptotic pathways [106].

Understanding the molecular mechanisms underlying drug resistance is crucial for developing effective therapeutic strategies for cancer patients. Researchers have explored various approaches to overcome drug resistance in cancer, such as combination therapy with other targeted agents or immunotherapy, developing new drugs that target alternative signaling pathways or bypass mechanisms, and identifying predictive biomarkers that can guide treatment selection and monitoring [107], [108].

### 3.2 PTEN loss and resistance to BRAF inhibitors in melanoma

In melanoma, a form of skin cancer, PTEN is frequently compromised by mutations or deletions. The loss of PTEN function disrupts the balance within the PI3K/AKT pathway, leading to uncontrolled activation and fostering an environment conducive to cancer development and progression. Studies have shown that PTEN loss is associated with poor survival in stage III melanoma, and genomic alterations in PTEN are linked to protein loss, potentially predicting intrinsic resistance to BRAF inhibitor therapy. Furthermore, PTEN loss has been described as a driving factor of an immune evasion mechanism that results in a lack of T-cell tumor infiltration [109]. For example, a study demonstrated that the loss of PTEN leads to resistance to BRAF inhibitors in melanoma cells by suppressing the expression of BIM. This finding underscores the impact of PTEN deficiency on the cellular response to BRAF inhibitors, shedding light on the intricate molecular mechanisms underlying resistance to melanoma treatment [110]. Furthermore, another study revealed that PERK plays a crucial role in mediating resistance to BRAF inhibition in melanoma, particularly in cases where PTEN function is impaired. This discovery sheds light on the intricate molecular pathways involved in conferring resistance to BRAF inhibitors in melanoma cells with dysfunctional PTEN and provides valuable insights into potential therapeutic targets for overcoming this resistance [111].

Therefore, understanding the intricate molecular mechanisms governing PTEN loss and its effect on BRAF inhibitor resistance is pivotal for optimizing melanoma treatment strategies. Identifying patients with melanomas harboring PTEN alterations allows clinicians to anticipate potential resistance to BRAF inhibitors and to consider alternative or combination therapies.

### 3.3 PTEN loss and resistance to BRAF inhibitors in thyroid cancer

In thyroid cancer, mutations in BRAF, particularly the V600E mutation, can lead to resistance to BRAF inhibitors and anaplastic transformation. These mutations may be primary (already present in the tumor) or secondary (acquired during treatment), thus bypassing BRAF blockade by thyroid carcinoma. The mechanisms of resistance to BRAF inhibitors in thyroid cancer include PIK3CA mutations that hyperactivate ERK when BRAF is inhibited [112]. Studies have shown that thyroid carcinomas bearing BRAF mutations are less sensitive to BRAF inhibitors and can develop primary or acquired resistance due to mutational events or the activation of alternative pathways [113]. Combination therapies involving BRAF inhibitors and other targeted agents are being explored to overcome drug resistance and enhance the efficacy of single-agent BRAF inhibitors [113].

Recent research has highlighted the role of fibronectin in the treatment of BRAF-mutant thyroid cancer. In resistant thyroid cancer cells treated with a BRAF inhibitor, fibronectin levels increased, promoting invasiveness and potential metastasis. Inhibiting ERK1/2 in the BRAF pathway decreases fibronectin secretion, and combining BRAF and ERK1/2 inhibitors slows tumor growth and reduces fibronectin levels [114].

PTEN loss in thyroid cancer can lead to resistance to BRAF inhibitors due to the interconnected nature of the PI3K/AKT pathway, which is regulated by PTEN, and the MAPK pathway, which is driven by mutant BRAF. This scenario creates a situation in which PTEN-deficient thyroid cancer cells may activate alternative survival pathways, such as PI3K/AKT, when treated with BRAF inhibitors. This compensatory activation diminishes the efficacy of BRAF inhibitors, limiting their ability to suppress tumor growth [115].

BRAF inhibitors induce feedback activation of the RAS pathway in thyroid cancer cells, potentially leading to drug resistance. Studies have investigated the effects of BRAF inhibitors such as vemurafenib and dabrafenib on cell growth and redifferentiation strategies in BRAFV600E mutated thyroid cancer cell lines. Combination therapies targeting multiple nodes of the MAPK pathway are being explored to improve outcomes and circumvent drug resistance in patients with advanced thyroid cancer treated with MAPK inhibitors [116].

Therefore, understanding the mechanisms of BRAF inhibitor resistance in thyroid cancer is crucial for developing effective treatment strategies and improving patient outcomes. Combination therapies and targeted approaches are being investigated to address drug resistance and enhance the efficacy of BRAF inhibitor treatment in patients with thyroid cancer.

## 4 *PTEN*-deficiency and immune checkpoint inhibitors resistance

### 4.1 PTEN loss and immune suppression in the tumor microenvironment

This section explores the intricate relationship between *PTEN* loss and resistance to immune checkpoint inhibitors (ICIs) in cancer therapy, specifically focusing on how *PTEN* deficiency contributes to immune system suppression within the tumor microenvironment.

ICIs are monoclonal antibodies that target inhibitory checkpoint molecules expressed by the membranes of antigen-presenting cells (APCs) and CD4 + T cells [117], [118]. ICIs have garnered attention in cancer therapy owing to their broad-ranging biological effects across diverse histological tumor types, their ability to elicit sustained responses, and their effectiveness in treating metastatic melanomas and overcoming resistance to chemotherapy. ICIs, exemplified by antibodies targeting programmed cell death protein 1 (PD-1), programmed cell death ligand 1 (PD-L1), and cytotoxic T-cell-associated protein 4 (CTLA-4), have revolutionized the field of cancer treatment [119], [120]. These agents unleash the full potential of the immune system by liberating T cells from inhibitory signals, thus enabling them to mount a potent anti-tumor response. However, the emergence of ICI resistance poses a formidable clinical challenge [121], [122]. ICIs have resulted in a fundamental shift in the clinical treatment of cancer. Nonetheless, the percentage of patients who can truly benefit from ICI therapy remains relatively limited despite the promising results observed in numerous cancer types through cancer immunotherapy [123]. Unavoidable issues include immune-related side effects and excessive expense [124].

**Fig. 3:**
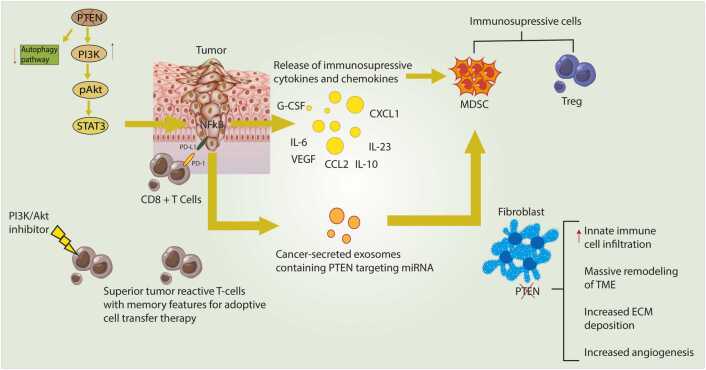
The role of PTEN in modulating the cancer immune microenvironment is significant. When PTEN is lost in tumor cells, it contributes to the creation of an immunosuppressive microenvironment. This environment is marked by the secretion of immunosuppressive cytokines and chemoattractants, which recruit MDSCs and Tregs. Furthermore, PTEN loss inhibits autophagy and diminishes the cytotoxicity of CD8 T cells. Additionally, tumor cells lacking PTEN release exosomes containing miRNAs that target PTEN expression in MDSCs, thereby promoting their activation.

Loss of *PTEN* in tumor cells has been shown to induce an immunosuppressive microenvironment. This is achieved through various mechanisms, including secretion of immunosuppressive cytokines and chemoattractant molecules that attract myeloid-derived suppressor cells (MDSCs) and regulatory T cells (Tregs). Additionally, *PTEN* loss inhibits the autophagy pathway, hinders CD8 + T cell cytotoxicity, and involves the release of miRNA-containing exosomes that target *PTEN* expression in MDSCs, thereby inducing their activation (Fig. 3) [125], [126]. The absence of *PTEN* in cancer cells plays a central role in augmenting resistance to ICIs by fostering immunosuppression within the tumor microenvironment [125], [127]. *PTEN* plays a crucial role in regulating the PI3K/Akt pathway, which is involved in various cellular processes, including cell growth, proliferation, and survival [128]. *PTEN* deficiency has been linked to the development of various cancers and is associated with a poor prognosis [128]. Recent research has shown that *PTEN* loss or mutation contributes to an immunosuppressive tumor microenvironment and poor response or resistance to immune checkpoint blockade therapy. Resistance to ICIs, driven by *PTEN* deficiency, unfolds through a network of complex mechanisms [129], [130]. In PTEN-deficient contexts, the PI3K/Akt pathway regulation is disrupted, resulting in uncontrolled signaling that promotes an immunosuppressive milieu [131], [132]. A recent study showed that reactivating PTEN promotes antitumor immunity by triggering an antitumor immune response in cancer cell lines by inducing autophagy activation and DAMP release [128]. In mouse models of PTEN-null prostate cancer and PTEN-treated melanoma, the administration of mPTEN@NP, a novel polymeric nanoparticle platform designed for PTEN mRNA delivery, resulted in the reduction of the immunosuppressive microenvironment within the tumor tissue. This alteration in the tumor microenvironment renders it more responsive to immune checkpoint blockade therapy [128].

In *PTEN*-deficient tumors, the release of immunosuppressive cytokines, such as TGF-β and IL-10, has been observed. These cytokines inhibit effector T cell function and promote the proliferation of Tregs, which suppresses the immune response. This impedes the activity of cytotoxic T cells and facilitates immune evasion [129]. Moreover, In *PTEN*-deficient tumors, MDSCs accumulation was observed In PTEN-deficient tumors. These cells exert potent immunosuppressive effects on T cells, further hindering the anti-tumor immune response [128], [133].

Recognizing the critical role of *PTEN* loss in driving ICIs resistance and fostering immunosuppression is essential for devising successful treatment strategies. Promising strategies involve combining ICIs with agents that target the PI3K/Akt pathway or modify the tumor microenvironment to overcome *PTEN*-related drug resistance. Profiling the *PTEN* status of patients before ICI treatment can serve as a predictive biomarker, enabling personalized therapies. Innovative immunomodulatory methods aim to counteract *PTEN* the immunosuppressive effects of PTEN loss and rekindle the anti-tumor immune response.

## 5 Cross-talk between *PTEN* and other signaling pathways

### 5.1 PTEN and PI3K/AKT signaling axis

*PTEN*, an estimated tumor suppressor, is a critical regulator of cellular fate. Its primary role is to counteract the PI3K/AKT signaling pathway and govern fundamental cellular processes, such as growth, survival, and apoptosis [134]. *PTEN's* mission involves dephosphorylating PIP3 into PIP2, thereby curtailing the unbridled growth-promoting signals orchestrated by PI3K/AKT [135].

In *PTEN*-deficient cancers, The PI3K/AKT pathway plays a pivotal role in PTEN-deficient cancers. The absence of *PTEN* relieves the restraint of PI3K, enabling uncontrolled phosphorylation of PIP2 into PIP3 [135]. Consequently, the downstream effector, AKT kinase, undergoes hyperactivation. This unleashes a chain of events including enhanced survival [136], unfettered proliferation [137], angiogenesis, metastasis, and therapy resistance [136].

Activation of the PI3K/AKT pathway in *PTEN*-deficient cancer cells often leads to resistance to multiple cancer treatments including chemotherapy, targeted therapies, and immunotherapy. Understanding the complex interplay between *PTEN* and the PI3K/AKT signaling pathway opens avenues for innovative strategies to combat cancer resistance. This includes exploring investigational PI3K and AKT inhibitors to target hyperactive signaling in *PTEN*-deficient cancers, combining these inhibitors with standard therapies or immunotherapy to enhance treatment effectiveness, and using *PTEN* profiling as a predictive biomarker to guide personalized treatment approaches.

### 5.2 PTEN and MAPK/ERK signaling interplay

The MAPK/ERK pathway is one of the best-defined pathways in cancer biology and regulates cellular proliferation, differentiation, and survival [138], [139]. Recent studies have revealed that AMPK signaling can reversibly modulate hyperactive MAPK signaling in cancer cells by phosphorylating key components, namely, RAF/KSR family kinases. This regulatory mechanism not only impacts the carcinogenic process but also influences the disease outcome [138]. The interplay between *PTEN* and the MAPK/ERK pathway is complex and can ultimately determine the cell fate. The intricate crosstalk between these pathways orchestrates a dynamic equilibrium within cells that can ultimately determine their fate [138].

The interaction between *PTEN* and MAPK/ERK signaling is pivotal for resistance mechanisms [140]. *PTEN* deficiency can disrupt the balance between the PI3K/AKT and MAPK/ERK pathways, potentially leading to feedback activation of MAPK/ERK and promoting cell survival and proliferation despite therapeutic interventions [141]. *PTEN*-deficient cancer cells may also switch to the MAPK/ERK pathway as an alternative survival route when PI3K/AKT signaling is blocked, conferring resistance to targeted therapies. Moreover, *PTEN* loss can trigger the upregulation of growth factor receptors and downstream molecules within the MAPK/ERK pathway, thereby strengthening cancer cell growth and reducing susceptibility to treatment [142].

Understanding the interplay between *PTEN* and MAPK/ERK signaling is key to the development of innovative therapies. Combining PI3K/AKT and MAPK/ERK inhibitors to combat *PTEN*-driven resistance, profiling *PTEN* status for personalized treatment selection, and advancing precision medicine by leveraging *PTEN*/MAPK insights for more effective cancer therapy.

### 5.3 PTEN and the Wnt/β-catenin signaling pathway in cancer

The intricate interplay between *PTEN* and Wnt/β-catenin signaling pathway adds layers of complexity to the narrative of cancer biology [143]. While *PTEN* primarily serves as an antagonist of PI3K/AKT signaling, its interactions with the Wnt/β-catenin pathway offer a complex dance of signaling cascades that can ultimately determine cell fate [144].

In the context of resistance mechanisms, the interplay between *PTEN* and Wnt/β-catenin signaling is a pivotal factor [145]. This intricate interaction manifests through several distinct mechanisms. For example, *PTEN* deficiency can lead to the stabilization and nuclear translocation of β-catenin, a key player in the Wnt pathway. This event can activate Wnt target genes, promoting cell proliferation and survival [146]. Dysregulation of Wnt/β-catenin signaling induced by *PTEN* loss may enhance cancer stem cell properties, fostering tumorigenesis and resistance to therapy [144], and *PTEN* deficiency may stimulate EMT through Wnt/β-catenin signaling, promoting metastasis and resistance to therapeutics [147].

A deep understanding of the *PTEN*-Wnt/β-catenin interplay offers innovative approaches: combining PI3K/AKT and Wnt/β-catenin inhibitors to counter *PTEN*-driven resistance, profiling PTEN status for personalized treatments, and advancing precision medicine through *PTEN*-Wnt/β-catenin insights to enhance the efficiency and outcomes of cancer therapy.

## 6 Emerging therapeutic strategies targeting *PTEN*-associated drug resistance

### 6.1 PTEN restoration approaches

*PTEN*, a highly regarded tumor suppressor, often causes mutations, deletions, or epigenetic silencing in the context of cancer, giving rise to drug resistance mechanisms [88], [148]. Consequently, PTEN restoration has emerged as an enticing strategy to counter this resistance.

One promising avenue involves gene therapy, which employs various techniques to introduce functional *PTEN* genes into cancer cells. Adenoviral vectors serve as vehicles for delivering intact *PTEN* genes, aiming to reinstate crucial *PTEN* functions in the cellular environment [149], [150]. Additionally, cutting-edge CRISPR-Cas9 technology offers precise gene-editing tools capable of repairing *PTEN* mutations or restoring *PTEN* expression, potentially reversing resistance mechanisms [129].

Advanced pharmacological agents are currently being investigated as potential *PTEN* restoration tools. Small molecules designed to mimic *PTEN’s* enzymatic activity of PTEN are currently being developed. These compounds compensate for *PTEN* loss by rebalancing aberrant signaling pathways. Furthermore, histone deacetylase (HDAC) inhibitors have been explored for their ability to reverse the epigenetic inactivation of *PTEN*, thereby restoring its tumor-suppressive expression and function [151].

Virus-mediated *PTEN* expression is an intriguing approach to *PTEN* restoration. Oncolytic viruses engineered to selectively infect and destroy cancer cells are armed with the PTEN gene [152], [153]. These viruses, such as adenoviruses, facilitate the targeted delivery and expression of *PTEN* in cancer cells, potentially reversing resistance mechanisms. Combination therapies offer a multi-pronged strategy to bolster *PTEN* restoration approaches. Combining inhibitors targeting the PI3K/AKT and Wnt/β-catenin pathways may prove effective in hindering the resistance mechanisms due to *PTEN* deficiency. Profiling the *PTEN* status of patients with cancer before initiating treatment can serve as a predictive biomarker, guiding the selection of tailored therapeutic approaches. Ongoing research endeavors are focused on developing precision medicine approaches that leverage insights into *PTEN* and Wnt/β-catenin signaling interactions to deliver more efficient and personalized cancer therapies [154].

Combination therapies that incorporate *PTEN* restoration strategies with standard treatments, such as chemotherapy or targeted therapies, show promise in overcoming drug resistance [155]. Furthermore, synergizing *PTEN* restoration with immunotherapy, particularly immune checkpoint inhibitors, could enhance the immune response within the tumor microenvironment. Routine *PTEN* status profiling in patients with cancer is crucial for personalized treatment approaches, which can significantly improve precision and effectiveness. Continued research is vital to refine *PTEN* restoration methods and address drug resistance in cancer, potentially revolutionizing cancer treatment by restoring *PTEN's* tumor-suppressive role of PTEN [156].

### 6.2 Combination therapies to overcome PTEN-related resistance

**Fig. 4:**
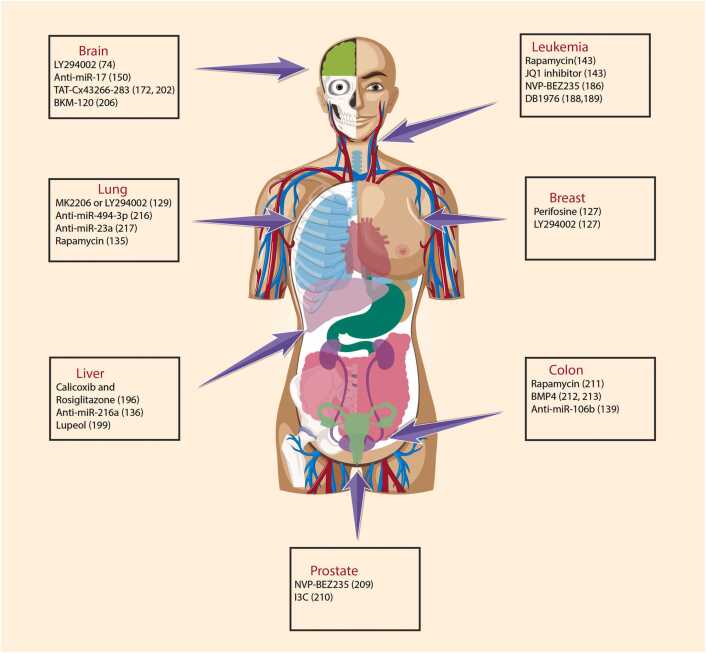
This figure outlines strategies targeting PTEN-mediated drug resistance in diverse cancers. In nervous system cancers like GBM and SHH-MB, PI3K/AKT inhibitors (e.g., LY294002, BKM-120) with chemotherapy inhibit tumors and glioma stem cells. Gliomas benefit from anti-miR-17 and TAT-Cx43266–283, elevating PTEN. Liver cancer responds to celecoxib and rosiglitazone, reducing AKT phosphorylation and increasing PTEN, impacting proliferation. Prostate cancer sees tumor regression with NVP-BEZ235 and chemotherapy. Leukemia interventions (rapamycin, NVP-BEZ235, JQ1, DB1976) reduce leukemic stem cells. In colon cancer, rapamycin with chemotherapy inhibits growth, while BMP4 up-regulates PTEN. Breast cancer responds to PI3K/AKT inhibitors (e.g., LY294002, perifosine). Lung cancer management involves LY294002, MK2206, and rapamycin inhibiting the PI3K/AKT/mTOR pathway. Anti-miRs (494–3p, 23a) prevent metastasis and restore chemotherapy sensitivity in LCSCs. These strategies aim to overcome drug resistance and suppress tumor growth across various cancers.

Combination therapies offer a multifaceted strategy to address the resistance mechanisms associated with *PTEN* (Fig. 4) [148]. Integrating *PTEN* restoration approaches with conventional cancer therapies, such as chemotherapy, offers promise for sensitizing cancer cells to treatment. Reinstating *PTEN* function alongside standard therapies makes it possible to counteract the resistance mechanisms stemming from *PTEN* deficiency. This combined approach can potentially enhance treatment efficacy, disrupt resistance pathways, and restore sensitivity to traditional cancer treatments [128].

Combining *PTEN* restoration with targeted therapies represents an innovative strategy to combat the resistance mechanisms associated with *PTEN* alterations [157]. Targeted therapies, which often focus on specific molecular pathways that drive cancer growth, can be complemented by *PTEN* restoration. Reinstating *PTEN* function can interfere with the signaling pathways that promote resistance to targeted agents. This combination approach can potentially enhance the effectiveness of targeted therapies and restore their ability to inhibit cancer progression [157].

The synergy between *PTEN* restoration and immunotherapy, particularly with immune checkpoint inhibitors, presents an intriguing avenue. *PTEN* plays a pivotal role in modulating immunosuppression in the tumor microenvironment. By restoring *PTEN* function, the efficacy of immunotherapy can be enhanced. *PTEN*-associated immunosuppression can be counteracted to promote a robust antitumor immune response. This combination approach holds promise for harnessing the immune system’s power to target and eliminate cancer cells [158].

Routine *PTEN* status profiling in patients with cancer is essential for personalized treatment and tailoring strategies to specific *PTEN* alterations to enhance accuracy and effectiveness. Biomarker-driven treatments ensure customized combination therapies that maximize success by considering the unique genetic and molecular characteristics of each tumor [159]. Ongoing research is crucial for optimizing combination therapies addressing *PTEN*-associated drug resistance, offering hope and potential for revolutionizing cancer treatment through these carefully designed approaches. The validation and enhancement of these strategies through continued research, clinical trials, and translational studies are imperative.

### 6.3 Precision medicine approaches based on PTEN status

Precision medicine represents a personalized treatment paradigm in which therapeutic strategies are tailored to the unique genetic and molecular characteristics of each patient [160], [161]. This approach can potentially revolutionize the management of *PTEN*-related resistance [162], [163]. Routine assessment of *PTEN* status in patients with cancer is imperative. This profiling encompasses comprehensive genetic and molecular analyses to identify *PTEN* alterations in PTEN, including mutations, deletions, or epigenetic silencing. This profiling allows patients to be categorized into distinct groups based on their *PTEN* status, thereby enabling personalized treatment strategies [159], [164].

Precision medicine harnesses the knowledge of *PTEN* status to select the most appropriate targeted therapies for individual patients. Patients with intact *PTEN* may benefit from targeted therapies that exploit specific molecular vulnerabilities within their tumors. Conversely, patients with *PTEN* alterations, such as loss or mutation, may receive therapies designed to counteract the consequences of *PTEN* deficiency [158], [165].

Precision medicine extends to the realm of combination therapies. *PTEN* status profile guides the selection of combination therapies that are most likely to be effective for each patient. For instance, patients with *PTEN* deficiency may benefit from a combination of *PTEN* restoration strategies and targeted therapies [166], [167].

Personalized Immunotherapy, including immune checkpoint inhibitors, can be personalized based on *PTEN* status. PTEN's pivotal role in modulating immunosuppression within the tumor microenvironment allows for the optimization of immunotherapeutic approaches to counteract *PTEN*-associated immunosuppression [130].

Implementing precision medicine approaches based on *PTEN* status represents a promising avenue for overcoming drug resistance associated with cancer-related *PTEN* alterations in PTEN. However, ongoing research, clinical trials, and translational research are essential to validate and optimize these strategies. As we navigate the complex landscape of *PTEN*-associated drug resistance, precision medicine offers hope and potential for a more personalized and effective approach to cancer treatment.

## 7 PTEN as a prognostic biomarker

**Fig. 5:**
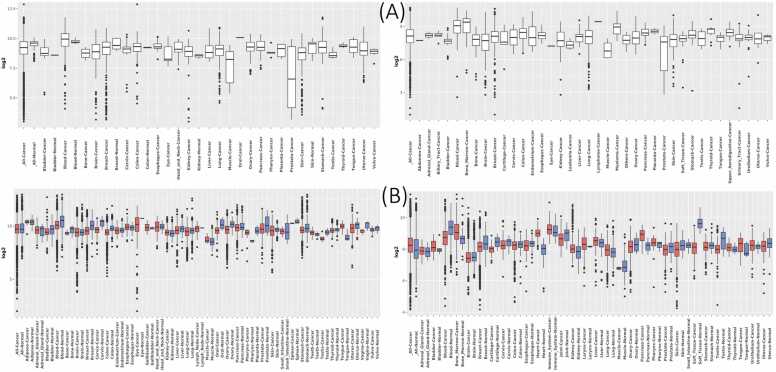
Comparison of PTEN expression levels between normal and cancer cells (A), and tissue (B). The figure was obtained from the Genet2 database.

Over the past few decades, there has been significant focus on the discovery of prognostic and predictive biomarkers. The primary objective is to enhance the precision of outcome predictions for individual patients and optimize treatment responses. Within this field of research, predictive biomarkers have emerged as pivotal elements in the development of cancer drugs. These biomarkers are of such significance that regulatory agencies have granted approval for dedicated tests to precisely measure them in conjunction with the approval of corresponding drugs. Consequently, these drugs can be prescribed exclusively to patients who test positive for relevant biomarkers, a practice commonly referred to as companion diagnostics [168], [169]. Despite its acknowledged significance in cancer biology and its potential relevance to the clinical behavior of tumors, the role of PTEN status as a prognostic and/or predictive biomarker remains a topic of ongoing debate. Currently, there are no specific and validated tests available to accurately assess the function or activity of PTEN. Fig. 5 shows the expression of PTEN in various cancer cells and tissues.

## 8 Clinical implications and future directions for targeting *PTEN*

PTEN status plays a pivotal role as a predictive biomarker for cancer. Assessing *PTEN* alterations before initiating therapy is imperative, as it can reveal the underlying resistance mechanisms and guide treatment decisions. To this end, clinical laboratories employ techniques such as immunohistochemistry (IHC) and genetic testing to provide valuable insights into *PTEN* status. These diagnostic tools enable clinicians to tailor treatment to individual patients based on *PTEN* profiles.

The development of effective strategies for *PTEN* restoration presents formidable challenges. Although promising approaches such as *PTEN* gene therapy, CRISPR-Cas9 technology, and small-molecule *PTEN* mimics exist, they require rigorous clinical validation. Tumor heterogeneity and resistance mechanisms must be addressed when developing targeted therapies to restore *PTEN* function. Combining *PTEN* restoration strategies with conventional treatments, targeted therapies, or immunotherapy offers a potential avenue to circumvent drug resistance.

The epigenetic silencing of *PTEN* presents complex challenges. Investigating HDAC inhibitors and other epigenetic modifiers may provide innovative solutions for reactivating *PTEN* expression. Furthermore, integrating *PTEN*-targeted therapies with immunotherapy provides opportunities to enhance immune responses. Research should focus on understanding the interplay between *PTEN* and the tumor microenvironment to optimize immunotherapeutic approaches.

Future clinical trials should prioritize biomarker-driven approaches, wherein patient stratification based on *PTEN* status informs treatment allocation. This strategy ensures that therapies are customized for patients that are most likely to benefit. Investigating combination strategies, such as *PTEN* restoration combined with immune checkpoint inhibitors or targeted therapies, holds promise in overcoming drug resistance and improving treatment outcomes.

Efforts to bridge the gap between laboratory findings and clinical applications are paramount. Adequate funding and support for translational research are essential to expedite the clinical translation of *PTEN*-targeted therapies. Adopting a patient-centered approach is of utmost importance, as we progress in understanding *PTEN*-mediated drug resistance. Incorporating patient preferences, values, and feedback into treatment decisions ensures holistic and personalized cancer care.

Exploration of cutting-edge technologies, including liquid biopsy and advanced imaging techniques, is imperative for noninvasive monitoring of *PTEN* status and assessment of treatment responses. In conclusion, *PTEN*-mediated resistance mechanisms in cancer pose challenges. While they present multifaceted challenges, they also provide fertile ground for innovative therapies and personalized, patient-centered care in the relentless battle against cancer.

## 9 Conclusion

In conclusion, the exploration of PTEN-mediated drug resistance mechanisms in cancer has revealed the complexity of this landscape, presenting both challenges and opportunities for innovation in cancer treatment. Our journey uncovered PTEN as a pivotal player, orchestrating critical cellular signaling pathways and perpetuating malignant cell growth through its alterations. With a focus on emerging therapeutic strategies, including PTEN restoration approaches and the promise of combination therapies, we anticipate a transformative potential in personalized cancer therapy. The paradigm of precision medicine, bolstered by PTEN status, is key to enhancing treatment efficacy and minimizing side effects. In the future, the imperative need for innovative strategies, biomarker-driven approaches, and patient-centric healthcare has become increasingly evident, offering hope and potential strides to overcome drug resistance and advance patient outcomes in the battle against cancer.

## Funding

Not applicable.

## CRediT authorship contribution statement

**Muhammad Tufail:** Writing – review & editing, Writing – original draft, Visualization, Validation, Software, Investigation, Data curation, Conceptualization.

## Declaration of Competing Interest

Not applicable.

## Data Availability

No data was used for the research described in the article.
